# Prospective Analysis of Traffic Exposure as a Risk Factor for Incident Coronary Heart Disease: The Atherosclerosis Risk in Communities (ARIC) Study

**DOI:** 10.1289/ehp.11290

**Published:** 2008-07-08

**Authors:** Haidong Kan, Gerardo Heiss, Kathryn M. Rose, Eric A. Whitsel, Fred Lurmann, Stephanie J. London

**Affiliations:** 1 Epidemiology Branch, National Institute of Environmental Health Sciences, National Institutes of Health, Department of Health and Human Services, Research Triangle Park, North Carolina, USA; 2 Department of Environmental Health, School of Public Health, Fudan University, Shanghai, China; 3 Department of Epidemiology, School of Public Health, University of North Carolina at Chapel Hill, Chapel Hill, North Carolina, USA; 4 Department of Medicine, School of Medicine, University of North Carolina at Chapel Hill, Chapel Hill, North Carolina, USA; 5 Sonoma Technology Inc., Petaluma, California, USA

**Keywords:** air pollution, coronary disease, traffic

## Abstract

**Background:**

For people living close to busy roads, traffic is a major source of air pollution. Few prospective data have been published on the effects of long-term exposure to traffic on the incidence of coronary heart disease (CHD).

**Objectives:**

In this article, we examined the association between long-term traffic exposure and incidence of fatal and nonfatal CHD in a population-based prospective cohort study.

**Methods:**

We studied 13,309 middle-age men and women in the Atherosclerosis Risk in Communities study, without previous CHD at enrollment, from 1987 to 1989 in four U.S. communities. Geographic information system–mapped traffic density and distance to major roads served as measures of traffic exposure. We examined the association between traffic exposure and incident CHD using proportional hazards regression models, with adjustment for background air pollution and a wide range of individual cardiovascular risk factors.

**Results:**

Over an average of 13 years of follow-up, 976 subjects developed CHD. Relative to those in the lowest quartile of traffic density, the adjusted hazard ratio (HR) in the highest quartile was 1.32 [95% confidence interval (CI), 1.06–1.65; *p*-value for trend across quartiles = 0.042]. When we treated traffic density as a continuous variable, the adjusted HR per one unit increase of log-transformed density was 1.03 (95% CI, 1.01–1.05; *p* = 0.006). For residents living within 300 m of major roads compared with those living farther away, the adjusted HR was 1.12 (95% CI, 0.95–1.32; *p* = 0.189). We found little evidence of effect modification for sex, smoking status, obesity, low-density lipoprotein cholesterol level, hypertension, age, or education.

**Conclusion:**

Higher long-term exposure to traffic is associated with incidence of CHD, independent of other risk factors. These prospective data support an effect of traffic-related air pollution on the development of CHD in middle-age persons.

Several prospective cohort studies suggest that long-term exposure to outdoor air pollution is associated with increased mortality from cardiopulmonary diseases (Abbey et al. 1999; Chen et al. 2005; Dockery et al. 1993; Filleul et al. 2005; Miller et al. 2007; Pope et al. 2002, 2004). Road traffic is a major contributor to outdoor air pollution in industrialized countries, contributing fine particulate matter (PM), carbon monoxide, oxides of nitrogen, and other pollutants. Assessment of traffic exposure can enhance studies of health effects of outdoor air pollution because local sources are important and because few people live close to the monitoring stations, which are often purposefully located away from local sources such as busy roads.

Recent studies have shown associations of long-term and short-term exposure to traffic air pollution with cardiovascular mortality, morbidity, and subclinical parameters (de Paula Santos et al. 2005; Finkelstein et al. 2004; Hoek et al. 2002; Hoffmann et al. 2006, 2007; Lanki et al. 2006; Peters A et al. 2004; Schwartz et al. 2005; Tonne et al. 2007; Volpino et al. 2004). In contrast, few prospective studies have examined traffic air pollution and coronary events. A recent study of survivors of myocardial infarction in Rome lacked information on smoking, an important potential confounder (Rosenlund et al. 2008). Two other prospective studies, one in Canada (Finkelstein et al. 2004) and the other in the Netherlands (Hoek et al. 2002), assessed only mortality. We need more prospective data on coronary events in healthy general populations, with detailed data on potential confounders, including smoking, collected at the individual level, to address the hypothesis that long-term traffic exposure influences the development of coronary heart disease (CHD).

In the present study, we examined the association between long-term residential traffic exposure and incident CHD events among participants in the Atherosclerosis Risk in Communities (ARIC) study, a prospective population-based cohort of middle-age men and women. This study included data on a wide range of risk factors for CHD collected prospectively at the individual level.

## Materials and Methods

### Participants

We studied participants from the ARIC study, which was designed to investigate the natural history and etiology of atherosclerosis and its sequelae. Details of the design, objectives, and quality control activities of the ARIC study have been previously reported (ARIC Investigators 1989). A probability sample of 15,792 residents 45–64 years of age was recruited in 1987–1989 from four U.S. communities: Forsyth County, North Carolina; Jackson, Mississippi; northwest suburbs of Minneapolis, Minnesota; and Washington County, Maryland. The Jackson sample was 100% African American, and the other three were predominantly white. The institutional review boards of the four participating centers approved the study, and all participants gave written informed consent before the study.

### Ascertainment of events

Study participants were followed for incident CHD until December 2002. Potential events were identified via annual telephone calls, community-wide hospital surveillance, and linkage with local and national death-certificate registries. We investigated events and deaths; we validated events using hospital records, and deaths using physician records and next-of-kin interviews. We defined incident CHD on the basis of published criteria as the first definite or probable myocardial infarction, silent myocardial infarction by electrocardiography, definite CHD death, or coronary revascularization (White et al. 1996). We classified events by a combination of computer algorithm and independent review by one or two physicians of medical record abstractions and discharge summaries.

### Geocoding

We geocoded participant addresses using a commercial service (Mapping Analytics LLC, Rochester, NY), which assigned a latitude and longitude coordinate to each address. Geocoding was performed with the Centrus Enhanced Database, which was primarily based on the Topologically Integrated Geographic Encoding and Referencing (TIGER) system data.

### Traffic exposure

We quantified small-scale spatial variations of traffic exposure by two measurements: geographic information system (GIS)–mapped traffic density assignments at place of residence, and the distance from place of residence to nearest roadways of various types. We used the participant’s address at the baseline visit (1987–1989) as the basis for calculating both exposure measures.

### Traffic density

We obtained the roadway locations and annual average daily traffic volumes from Geographic Data Technology (GDT; now Tele Atlas Global Crossroads, Boston, MA). We selected GDT roadway geometry data because they provide 100% roadway coverage in the four communities and are the most extensively georeferenced (or repositioned), using aerial imagery to match real-world locations. It is estimated that most GDT roads in populated areas are located with ±12 m “position accuracy.” GDT bases the traffic volumes on state and county agency traffic counts on highways, arterials, and collector streets with more than approximately 1,000 vehicles per day. They assign traffic counts to neighboring roadway links with similar capacity.

We used the link-based traffic volumes to generate maps of traffic density with 10 × 10 m resolution using the ARCInfo Spatial Analyst software (Kan et al. 2007; Peters JM et al. 2004). We created traffic density maps with 300 m circular search radii that produce densities decreasing by approximately 90% between the edge of the roadway and 300 m away (perpendicular) from the roadways, which is consistent with the characteristics observed by Zhu et al. (2002, 2006). We used identical mapping procedures in all the communities so that the results are comparable across communities. The densities reflect proximity to traffic without consideration of differential exposures caused by meteorology. This method accounts for the combined relative influence of several roadways (and road types) with various traffic activity levels at different distances from each residence location. This metric generally behaves like an inverse-distance–weighted traf-fic volume, except that it specifically considers intersections and multiple roadways more accurately. Therefore, these density values provide a relative indication of which residence locations are likely to be most exposed to traffic activity and, as such, are dimensionless indicators of proximity to traffic volume.

Because the available traffic density data were from 2000, we back-extrapolated to the study period (1987–1992) based on change in population density using county-level census population data. Changes in traffic volumes over time are correlated with changes in population density (Polzin 2006).

### Distance to major roads

To estimate qualitatively the distribution of the distance from residence locations to roadways, we calculated straight-line distances. The distance-to-roadway data include the distance (in meters) from each unique residence location to the nearest roadways.

In a previous study, the concentrations of ultrafine PM from highway traffic became indistinguishable from the background concentration at distances > 300 m (Zhu et al. 2002). We therefore dichotomized distance to major roads (interstate and state highways, major arterials) at 300 m. To conduct analysis of sensitivity of the results to the choice of cut-points, we also categorized distance to major roads as ≤ 150 m and > 150 m (Hoffmann et al. 2006; Venn et al. 2001).

### Background air pollution level

We acquired data on the background ambient concentrations of PM with aerodynamic diameter ≥ 10 μm (PM_10_), nitrogen dioxide, and ozone during the research period from the U.S. Environmental Protection Agency air quality data retrieval system. We abstracted 24-hr average concentrations for PM_10_ and NO_2_ and 8-hr (from 1000 hours to 1800 hours) average concentrations for O_3_. We spatially interpolated the average concentrations from air quality monitoring stations to the cohort residence locations using inverse distance weighting.

### Other covariates

We defined hypertension as systolic blood pressure of ≥140 mmHg, diastolic blood pressure of ≥90 mmHg, or use of antihypertensive medication during the previous 2 weeks. We defined diabetes mellitus as a fasting glucose level of ≥126 mg/dL (7.0 mmol/L), a nonfasting glucose level of ≥200 mg/dL (11.1 mmol/L), or a self-reported history of or treatment for diabetes. Trained, certified technicians determined anthropometric measures following a detailed, standardized protocol. We calculated body mass index (BMI) as weight (kg)/[height (m)]^2^. Blood collection and processing for levels of total cholesterol, low-density lipoprotein (LDL), and high-density lipoprotein (HDL) are described elsewhere (National Heart, Lung, and Blood Institute 1988). Trained and certi-fied interviewers also collected information on age, ethnicity, sex, smoking, environmental tobacco smoke (ETS), alcohol consumption status, occupation, education, family income, and family history of CHD. We calculated the family risk score based on the participant’s report of parents and five oldest siblings’ history of CHD (Li et al. 2000). Smoking variables included smoking status (never, former, and current smokers), age at starting to smoke, years smoked, and cigarettes per day. We classified never smokers and former smokers as exposed to ETS if they reported being in close contact with smokers for more than 1 hr/week (Howard et al. 1998). Thus, we obtained five strata for active and passive smoking: current smoker, former smoker with ETS, former smoker without ETS, never smoker with ETS, never smoker without ETS. Neighborhood-level socioeconomic status (SES), in addition to individual level factors, may affect health status (Geronimus and Bound 1998), so we included 1990 census-tract–level data on median employment rate and poverty rate (U.S. Census Bureau 1992).

### Statistical analysis

The end point of interest was incident CHD, so we excluded participants if they had prevalent CHD (*n* = 762). We also excluded persons who met the following criteria: ethnicity other than African American or white (*n* = 48) and, because of their small numbers, African Americans from Minnesota and Maryland field centers (*n* = 55); and missing geocoding information (*n* = 1,724). Exclusions overlapped in some instances, leaving 13,309 subjects for analysis.

We conducted all analyses using the statistical software package SAS, version 9.1 (SAS Institute Inc., Cary, NC). We calculated follow-up time as the time from baseline to an event, or the last follow-up contact, or through December 2002, whichever occurred first.

**Figure 1:**
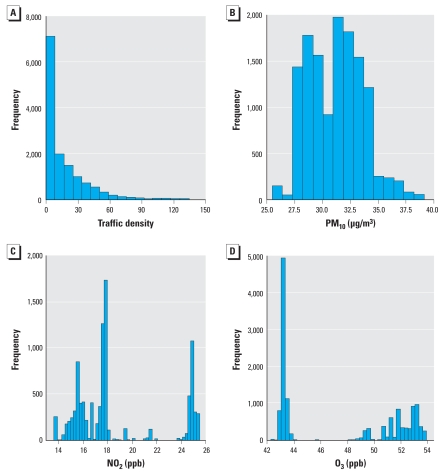
Distribution of traffic density at ARIC participant residences (1987–1989). (*A*) Traffic density (*n* = 13,309). (*B*) PM_10_ ( μg/m^3^; *n* = 13,309). (*C*) NO_2_ (ppb; *n* = 9,902). (*D*) O_3_ (ppb; *n* = 13,309).

We used Cox proportional hazards regression analyses to assess the associations of traffic exposure with the risk of incident CHD. Distributions of traffic density are highly skewed (Figure 1); therefore, we analyzed traf-fic density both as quartiles and as a continuous variable after log transformation. We estimated the hazard ratios (HRs) of incident CHD for quartiles of traffic density relative to the lowest quartile, and for one unit increase of log-transformed density values. For tests for linear trends across increasing quartiles of traf-fic density, we used the median value in each quartile. We also estimated the risk for living close to major roads (≤300 m or ≤150 m), using living farther away as the reference.

Our basic models included age, sex, center, and ethnicity (Miller et al. 2007). In the adjusted models, we added factors that we iden-tified *a priori* as potential confounders: BMI, physical activity, education, occupation, individual family income, census-tract–based SES (median employment rate and poverty rate), smoking status (current smoker, former smoker with ETS, former smoker without ETS, never smoker with ETS, never smoker without ETS), age at starting to smoke (0–15, 15–20, 20–29, and ≥30 years), years smoked, cigarettes per day, alcohol intake (never, former, and current drinker), hypertension, diabetes status, family risk score, HDL, LDL, total cholesterol, fib-rinogen, and background air pollution level (PM_10_ and O_3_). NO_2_ data were missing in Jackson (Figure 1), so we did not include them in the adjusted models. Because other concomitants of traffic exposure, such as stress, could affect cardiovascular health (Williams et al. 2000), we did a sensitivity analysis to examine the impact of social stress (trait anger), measured at the second cohort examination, on the estimated effect of traffic exposure.

We also conducted stratified analyses by sex, smoking status, obesity, LDL level, hypertension, age, and education, to examine potential modifiers of the association between traffic exposure and incident CHD. We categorized BMI according to the standard definition: normal/underweight (BMI < 25) and overweight/obese [BMI ≥25 (Centers for Disease Control and Prevention 2007)]. We dichotomized LDL level as ≤130 mg/dL and > 130 mg/dL. We defined hypertension as systolic blood pressure of ≥140 mmHg, diastolic blood pressure of ≥90 mmHg, or use of anti-hypertensive medication during the previous 2 weeks. We classified education as low (less than high school), middle (high school or vocational school), or high (college or above).

## Results

Table 1 presents selected characteristics of participants at baseline, according to whether or not they developed incident CHD during follow-up. Among the 13,309 study participants who were free of CHD at baseline, 976 subjects developed CHD (268 fatal and 708 non-fatal) over an average of 13 years of follow-up. As expected, subjects with incident CHD were slightly older; more likely to be male, black, or current smokers; had higher BMI, total cholesterol, and LDL and lower HDL; and had higher prevalence of hypertension and diabetes.

The estimated traffic density and back-ground air pollutant (PM_10_, NO_2_, and O_3_) concentrations at the baseline home address varied greatly (Figure 1). Consistent with previous reports (Hoek et al. 2001), we did not find a strong correlation between traffic density and background air pollution level; the Pearson correlation coefficients of traffic density with PM_10_, NO_2_, and O_3_ were –0.12, –0.04, and –0.10, respectively. Background PM_10_ was moderately correlated with NO_2_ (Pearson correlation coefficient, *r* = 0.60) and O_3_ (*r* = 0.44); NO_2_ was weakly correlated with O_3_ (*r* = 0.03).

**Figure 2:**
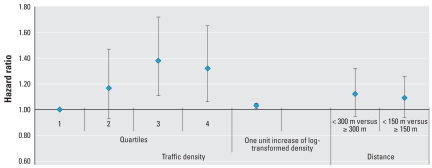
Adjusted HRs (and 95% CIs) for incident CHD in relation to traffic density and by distance to major roads. Covariates were age, sex, center, ethnicity, BMI, physical activity, education, occupation, individual family income, census-tract–based SES, smoking status, age at starting to smoke, years smoked, cigarettes per day, alcohol intake, hypertension, diabetes status, family risk score, HDL, LDL, total cholesterol, fibrinogen, and background air pollution level.

Greater traffic density was associated with increased risk of incident CHD in both basic and adjusted models (Table 2). Relative to those in the lowest quartile of traffic density, the adjusted HRs across increasing quartiles were 1.17 [95% confidence interval (CI), 0.93–1.47], 1.38 (95% CI, 1.11–1.72), and 1.32 (95% CI, 1.06–1.65) (*p*-value for trend across quartiles = 0.042). When we treated traffic density as a continuous variable, the adjusted HR per one unit increase of log-transformed density was 1.03 (95% CI, 1.01–1.05; *p* = 0.006) (Figure 2).

For residents living within 300 m of major roads compared with subjects living farther away, the adjusted HR (model 2) was 1.12 (95% CI, 0.95–1.32; *p* = 0.189) for incident CHD (Table 3, Figure 2). In the analysis with alternative cut point of distance to major roads (150 m), we found similar patterns with incident CHD (Table 3, Figure 2).

We further examined whether sex, smoking status, BMI, LDL level, hypertension, age, and education modified the association of traffic density with incident CHD (Table 4). Although results did not always achieve statistical significance with the reduced sample sizes in subgroup analyses, there were positive associations in most strata and little evidence of effect modification.

Consistent with the previous study of Hoek et al. (2002), we did not observe signifi-cant effects of background air pollution on incident CHD; the HRs of incident CHD per 10- μg/m^3^ increase of PM_10_ and O_3_ were 1.28 (95% CI, 0.76–2.18) and 1.04 (95% CI, 0.45–2.42), respectively. In addition, the observed associations between traffic and CHD remained after we further adjusted for trait anger (data not shown).

## Discussion

Among adults from four U.S. communities followed prospectively over an average of 13 years, higher residential exposure to traffic, a major source of air pollution in urban areas, was associated with an increased risk of incident CHD events. To our knowledge, our study provides the first prospective evidence of the association between traffic exposure and incident cardiovascular morbidity in the general population.

Traffic emissions result in small-scale spatial variations and therefore mainly affect residents living close to busy roads (Roorda-Knape et al. 1999). Thus, air pollution data from fixed monitoring stations may be inadequate to study traffic-related air pollution and health outcomes, especially for those living near busy roads. For example, Hoek et al. (2002) identi-fied a consistent association of cardiopulmonary mortality with traffic exposure, but not with estimated ambient background concentration of the traffic indicator pollutants black smoke and NO_2_. Similarly, we did not observe a significant effect of background air pollution on incident CHD. This is not surprising given that the ARIC study was not designed to examine air pollution and was conducted only in four communities. Furthermore, these four communities were not well supplied with air pollution monitors during the study period, resulting in little variation in measured air pollution within communities. In addition, from an analytic perspective, any affect of baseline air pollution on risk would be indistinguishable from differences in risk by community, a design variable for this study.

Our prospective results support previous findings from cross-sectional, case–control, and cohort studies examining the association between long-term traffic exposure and cardiovascular morbidity, mortality, or intermediate end points. In a prospective cohort analysis of myocardial infraction survival, Rosenlund et al. (2008) found that long-term exposure to traffic-related air pollution increased the risk of CHD, and the relative risk for incident coronary events per 10 μg/m^3^ of NO_2_ was 1.03 (95% CI, 1.00–1.07). In a case–control analysis in Boston, Massachusetts, an interquartile range increase in cumulative traffic near the home was associated with a 4% (95% CI, 2–7%) increase in the odds of acute myocardial infarction, suggesting an effect of long-term exposure to traffic (Tonne et al. 2007). In a cross-sectional analysis, Hoffmann et al. (2006) found that higher long-term exposure to traffic-related emission, but not background air pollution, was associated with increased risk of CHD events (odds ratio = 1.85; 95% CI, 1.21–2.84) in a German population. In the same study, residents living within 50 m from a major road had an odds ratio of 1.63 (95% CI, 1.14–2.33), relative to subjects living > 200 m away, for elevated coronary artery calcifica-tion, an intermediate cardiovascular end point (Hoffmann et al. 2007).

In the present study, we examined the association between traffic exposure at the baseline residences (visit 1, 1987–1989) and incident CHD. We found similar associations of traffic with CHD when we used the first-year (1987) exposure data. This is comparable with previous studies of air pollution in relation to mortality that assessed exposure at the beginning of follow-up (Hoek et al. 2002; Pope et al. 2002, 2004). Although air pollution levels may vary over time because of changes in emission or economic activity, substantial changes are usually slow and affect the region in the same way.

Various factors may modify the health effects of air pollution. We did not find signifi-cant evidence for effect modification by sex, smoking status, obesity, LDL cholesterol level, hypertension, age, or education. The information on modification of long-term effects of air pollution by educational status is inconsistent (Hoffmann et al. 2006; Pope et al. 2002). Additional examination of modifying factors in future investigations will help in public policy making, risk assessment, and standard setting.

Some limitations of our analysis should be noted. We did not predict the air pollutant concentration based on traffic density data, and we could not validate our exposure assessment with actual measurements given that the exposure period was 1987–1989. Likewise, our traffic density metric does not reflect the local meteorologic conditions that could influence the emission, mixing, and transport of air pollutants. Some studies have suggested a stronger association with stop-and-go traffic than with moving traffic and with truck traffic compared with car traffic (Ryan et al. 2005). However, in most studies, including ours, it was not possible to separate traffic types. As in some other studies, our exposure assessment was limited to residential address, and we lacked information on home exposures to other sources of pollutants, such as cooking or heating. Because our study outcomes are CHD events, rather than a measure of CHD pathogenesis, it is possible that some of the CHD events may be attributable to short-term exposure to traffic (Lanki et al. 2006; Peters A et al. 2004). However, given evidence that the association for acute exposure to air pollution is smaller in magnitude than the associations for long-term exposure (Künzli et al. 2005), we suspect that any overestimation of effects of long-term exposure to traffic would be minimal. Also, as in any epi-demiologic study, residual confounding is possible. However, we carefully adjusted for known and potential confounders, including demographic characteristics, personal and neighborhood level socioeconomic characteristics, cigarette smoking, family risk factors, and background air pollution.

We are not able to rule out the possible effect of traffic noise, which at high levels may have adverse effects on cardiovascular physiology (Berglund et al. 1996). However, the associations between traffic noise and cardiovascular risk are far less consistent than those between air pollution and cardiovascular disease (Ising and Kruppa 2004). Moreover, given that noise-related cardiovascular events (e.g., hypertension) may follow a different pathway than does air pollution (Jarup et al. 2008), and we found a significant association between traffic and CHD in nonhypertensive subjects (Table 4), it seems unlikely that noise explains the observed effects of traffic.

We lacked assessment of traffic-related air pollution for the approximately 10.9% of subjects whose addresses could not be geocoded. Most of the missing geocodes were attributed to such problems as missing state (most often military addresses), address left blank, temporary address, address not in the United States, apartment name without address, or only a post office box given. This could raise concern about potential selection bias. However, for missing geocode data to have created a spurious association between higher traffic exposure and incident CHD, subjects with and without geocodes would need to differ in both traffic exposure and incident CHD. Although we have no data on their traffic exposure, they were similar to subjects with nonmissing geocodes in sociode-mographic characteristics and incident CHD [see Supplemental Material, Table S1 (online at http://www.ehponline.org/members/2008/11290/suppl.pdf)]. Thus, it is unlikely that the missing geocode data could have created the observed associations.

Because we obtained the geocodes for participants’ addresses from the TIGER file by Mapping Analytics, error could result from the use of an older road network data. To assess this, we randomly selected 100 participants from each of the ARIC communities and regeocoded their residential addresses using the GDT software, which incorporates a more recent road network database. Using these new geocodes, we recalculated the traffic densities and distances to major roads and compared them with the original results. The two geocoding methods resulted in similar estimates for the distance to nearest major roads. For traffic density, the two methods yielded quite concordant values for the Forsyth, Jackson, and Minneapolis communities, but concordance was lower for Washington County. This might reflect a renaming of streets that occurred there, so we repeated our analyses excluding Washington County from our analysis. With the less precise exposure assessment in Washington County excluded, the association of traffic density with incident CHD remained [see Supplemental Material, Table S2 (online at http://www.ehponline.org/members/2008/11290/suppl.pdf)], suggesting that the association is relatively robust to geocoding error.

A major strength of our study is the use of an objective measure of traffic-related air pollution at residential addresses (e.g., GIS-based assessment of traffic density and distance to major roads) to capture exposure relevant for subjects living in close proximity to busy roads. A further strength of our study is the overall residential stability of the cohort; approximately 90% of the ARIC participants had lived in the same community for > 10 years at the baseline visit. An earlier study of ARIC study participants reported very high concordance between past decades and visit 1 for county and state of residence (Rose et al. 2004). Moreover, we based our analysis on carefully collected incidence data in a large cohort from four U.S. communities. We prospectively collected data on exposure, outcome, and a wide range of potential confounders at the individual levels using standardized protocols and extensive quality assurance. In addition to doing detailed adjustment for individual-level confounders and evaluating potential effect modification, we also adjusted for a community-level measure of SES to help account for confounding (Marmot 2001).

In summary, in this prospective analysis, higher long-term exposure to traffic, a major source of air pollution, was related to increased risk of incident CHD. These find-ings add to the previous data on mortality and disease prevalence and suggest that traffic-related air pollution can influence the development of disease in an ostensibly healthy middle-age population. Continued emphasis on the implementation of strategies for reducing traffic-related air pollution is likely to reap additional public health benefits.

## Figures and Tables

**Table 1:** Baseline characteristics of the ARIC participants by the status of incident CHD at end of follow-up (*n* = 13,309).^a^

Characteristic	Incident CHD (*n* = 976)	No incident CHD (*n* = 12,333)
Sex (% male)	59.3	41.4
Age (years)	55.8 ± 5.6	53.9 ± 5.8
BMI (kg/m^2^)	28.6 ± 5.4	27.6 ± 5.4
Ethnicity (% black)	31.4	28.4
Smoking (%)		
Current	38.7	25.1
Former	31.1	31.5
Never	30.0	43.4
Drinking (%)		
Current	49.7	57.2
Former	25.4	17.2
Never	24.7	25.6
Hypertension (%)	54.1	34.3
Diabetes (%)	28.4	10.0
Total cholesterol (mmol/L)	5.8 ± 1.2	5.5 ± 1.1
HDL (mmol/L)	1.2 ± 0.4	1.4 ± 0.4
LDL (mmol/L)	3.9 ± 1.1	3.5 ± 1.0

a Values are mean ± SD unless specified as percentage.

**Table 2:** HRs (95% CIs) for incident CHD associated with traffic density.

	Quartile					Continuous variable (log-transformed)	
Model	1 (lowest)	2	3	4	*p*-Value for trend^a^	One unit increase	*p*-Value
Median of quartiles	0	2.87	14.97	41.83			
Cases	223	228	262	263			
Basic model^b^	1.00	1.13 (0.94–1.37)	1.31 (1.09–1.57)	1.28 (1.07–1.54)	0.018	1.02 (1.01–1.04)	0.004
Adjusted model^c^	1.00	1.17 (0.93–1.47)	1.38 (1.11–1.72)	1.32 (1.06–1.65)	0.042	1.03 (1.01–1.05)	0.006

a *p*-Values for trend based on quartiles scaled by the quartile medians.

b Covariates were age, sex, center, and ethnicity.

c Covariates were age, sex, center, ethnicity, BMI, physical activity, education, occupation, individual family income, census-tract–based SES, smoking status, age at starting to smoke, years smoked, cigarettes per day, alcohol intake, hypertension, diabetes status, family risk score, HDL, LDL, total cholesterol, fibrinogen, and background air pollution level.

**Table 3:** HRs (95% CIs) for incident CHD by distance to major roads.

	Dichotomized at 300 m			Dichotomized at 150 m		
Model	< 300 m	≥300 m	*p*-Value	< 150 m	≥150 m	*p*-Value
Cases	683	293		408	568	
Basic model^a^	1.13 (0.98–1.30)	1.00	0.085	1.12 (0.99–1.28)	1.00	0.073
Adjusted model^b^	1.12 (0.95–1.32)	1.00	0.189	1.09 (0.94–1.26)	1.00	0.264

a Covariates were age, sex, center and ethnicity.

b Covariates were age, sex, center, ethnicity, BMI, physical activity, education, occupation, individual family income, census-tract–based SES, smoking status, age at starting to smoke, years smoked, cigarettes per day, alcohol intake, hypertension, diabetes status, family risk score, HDL, LDL, total cholesterol, fibrinogen, and background air pollution level.

**Table 4:** Adjusted HRs for incident CHD associated with traffic density, stratified by sex, smoking status, BMI, and education.^a^

		Higher traffic density		
Characteristic	No. (%) cases	Adjusted ratio^b^	*p*-Value for trend^c^	*p*-Value for interaction^d^
Sex				
Female	397 (5.2)	1.27 (0.91–1.78)	0.066	0.397
Male	579 (10.2)	1.41 (1.04–1.91)	0.138	
Smoking status				
Never	293 (5.2)	0.84 (0.56–1.24)	0.785	0.380
Current/former	681 (8.9)	1.61 (1.22–2.12)	0.013	
BMI				
< 25	254 (5.7)	1.49 (0.98–2.28)	0.051	0.271
≥25	719 (8.2)	1.29 (0.99–1.68)	0.208	
LDL (mg/dL)				
≤130	320 (5.5)	1.38 (0.93–2.03)	0.133	0.567
> 130	618 (8.8)	1.29 (0.98–1.70)	0.171	
Hypertension				
No	443 (5.2)	1.45 (1.04–2.02)	0.041	0.659
Yes	528 (11.1)	1.30 (0.96–1.77)	0.213	
Baseline age (years)				
≤60	733 (6.7)	1.36 (1.05–1.75)	0.028	0.624
> 60	243 (10.3)	1.10 (0.68–1.78)	0.991	
Education				
Low	321 (10.7)	1.09 (0.73–1.64)	0.725	0.705
Middle	372 (6.9)	1.74 (1.20–2.52)	0.016	
High	283 (5.8)	1.22 (0.80–1.86)	0.420	

a Covariates were age, sex, center, ethnicity, BMI, physical activity, education, occupation, individual family income, census-tract–based SES, smoking status, age at starting to smoke, years smoked, cigarettes per day, alcohol intake, hypertension, diabetes status, family risk score, HDL, LDL, total cholesterol, fibrinogen, and background air pollution level.

b Comparing the fourth with the first quartiles of traffic density (95% CI).

c *p*-Value for trend based on quartiles scaled by the quartile medians.

d *p*-Value for interaction between traffic exposure and stratification factors.
